# Linoleic Acid Induces Metabolic Reprogramming and Inhibits Oxidative and Inflammatory Effects in Keratinocytes Exposed to UVB Radiation

**DOI:** 10.3390/ijms251910385

**Published:** 2024-09-26

**Authors:** Carolina Manosalva, Claudio Bahamonde, Franco Soto, Vicente Leal, César Ojeda, Carmen Cortés, Pablo Alarcón, Rafael A. Burgos

**Affiliations:** 1 Institute of Pharmacy, Faculty of Sciences, Universidad Austral de Chile, Valdivia 5090000, Chile; 2 Laboratory of Inflammation Pharmacology and Immunometabolism, Institute of Pharmacology and Morphophysiology, Faculty of Veterinary Sciences, Universidad Austral de Chile, Valdivia 5090000, Chilerburgos1@uach.cl (R.A.B.)

**Keywords:** linoleic acid, keratinocyte, photodamage

## Abstract

Linoleic acid (LA), the primary ω-6 polyunsaturated fatty acid (PUFA) found in the epidermis, plays a crucial role in preserving the integrity of the skin’s water permeability barrier. Additionally, vegetable oils rich in LA have been shown to notably mitigate ultraviolet (UV) radiation-induced effects, including the production of reactive oxygen species (ROS), cellular damage, and skin photoaging. These beneficial effects are primarily ascribed to the LA in these oils. Nonetheless, the precise mechanisms through which LA confers protection against damage induced by exposure to UVB radiation remain unclear. This study aimed to examine whether LA can restore redox and metabolic equilibria and to assess its influence on the inflammatory response triggered by UVB radiation in keratinocytes. Flow cytometry analysis unveiled the capacity of LA to diminish UVB-induced ROS levels in HaCaT cells. GC/MS-based metabolomics highlighted significant metabolic changes, especially in carbohydrate, amino acid, and glutathione (GSH) metabolism, with LA restoring depleted GSH levels post-UVB exposure. LA also upregulated PI3K/Akt-dependent GCLC and GSS expression while downregulating COX-2 expression. These results suggest that LA induces metabolic reprogramming, protecting against UVB-induced oxidative damage by enhancing GSH biosynthesis via PI3K/Akt signaling. Moreover, it suppresses UVB-induced COX-2 expression in HaCaT cells, making LA treatment a promising strategy against UVB-induced oxidative and inflammatory damage.

## 1. Introduction

The skin, the body’s largest organ, serves as a protective barrier against harmful stimuli, including ultraviolet (UV) radiation from the sun. Although UVB radiation comprises only 4% to 5% of total solar emissions, it is the main contributor to skin damage, particularly within the 280–320 nm wavelength range [1,2,3]. UVB is a major factor in sunburn, leading to symptoms such as redness (erythema), inflammation, and pain. Unlike UVA, UVB has higher energy but penetrates less deeply, primarily affecting the basal cell layer of the epidermis, where it directly damages DNA [3]. UVB radiation produces reactive oxygen species (ROS), the central regulator of UVB-induced photodamage [4]. Although ROS have essential physiological functions in cell signaling, excessive levels can be toxic unless ROS are removed promptly. Keratinocytes are the most abundant cells in the epidermis and are most exposed to UVB radiation. Therefore, keratinocytes depend on efficient antioxidant mechanisms to maintain their function. Among these, the reduced form of glutathione (GSH) prevails as the primary cytoplasmic antioxidant, representing the most abundant thiol in animal cells [5]. Recent research conducted by Li et al. has demonstrated that UVB radiation induces metabolic rewiring in keratinocytes, affecting various metabolic pathways, including arginine and proline metabolism, the tricarboxylic acid (TCA) cycle, purine metabolism, the malate–aspartate shuttle, the urea cycle, and ammonia recycling [6]. Additionally, UVB radiation led to the downregulation of glycolysis, the TCA cycle, and fatty acid β-oxidation during the early stages of skin carcinogenesis in a murine model using UVB radiation [7]. Moreover, research has indicated that glutathione metabolism pathways are pivotal in the cellular dysregulation resulting from oxidative damage induced by UVB radiation in fibroblasts [8].

The altered GSH metabolism and resultant redox imbalance induced by UVB exposure could contribute to photoaging within this framework. This process is characterized by the accumulation of DNA damage, heightened ROS levels, and the subsequent activation of inflammatory responses along with cell cycle arrest [8]. However, the precise mechanisms through which UVB radiation affects GSH metabolism remain elusive; specific data have suggested a reduction in the enzymatic activity of GCLC (glutamate–cysteine ligase catalytic subunit) and GSS (glutathione synthetase), pivotal enzymes in GSH biosynthesis [9]. Hence, compounds capable of stimulating the expression of GCLC and GSS, as enhancers of GSH synthesis, have garnered escalating interest in treating various diseases driven by oxidative stress [10]. ROS production triggers the activation of mitogen-activated protein kinase (MAPK) signaling and the redox-sensitive transcription factor NF-κB (nuclear factor kappa light chain enhancer of activated B cells) [11], which orchestrates the transcription of pro-inflammatory genes. GSH inhibits NF-κB activation by curtailing ROS production and modulating the UV radiation-induced pro-inflammatory cytokine storm [12]. Consequently, it is imperative to investigate and develop effective pharmacological agents capable of restoring GSH metabolism, thereby reinstating the cellular redox equilibrium in UVB-exposed cells.

Linoleic acid, the primary 18-carbon ω-6 polyunsaturated fatty acid (PUFA) found in the healthy epidermis, plays a pivotal role in preserving the skin’s water permeability barrier [13,14,15]. Notably, dietary deficiency in LA induced a scaly, pruritic skin disorder alike to atopic dermatitis in hairless mice [16]. Recent research on vegetable oils rich in LA has unveiled their antioxidant and anti-inflammatory properties, indicating their potential to treat conditions with a prominent inflammatory component [17,18]. Furthermore, linoleic acid-enriched vegetable oils have been shown to significantly mitigate UV radiation-induced effects such as ROS production, cellular damage, and skin aging [19,20]. Nevertheless, the precise mechanisms underlying these effects of LA remain incompletely understood [21]. Recent findings have demonstrated that linoleic acid enhances antioxidant capacity through activating the adenosine monophosphate-activated protein kinase (AMPK) signaling pathway and the AMPK–target of rapamycin (TOR) signaling pathway in hepatocytes [21]. Moreover, this research revealed that the antioxidant effect of linoleic acid is primarily attributed to the stabilization of Nrf2 (nuclear factor erythroid 2-related factor 2), which orchestrates the antioxidant response and regulates the transcription of enzymes involved in glutathione synthesis [21]. Despite the established importance of LA for maintaining proper skin function, a comprehensive understanding of its mechanisms and roles in photoaging and the inflammatory response triggered by UVB radiation is still required.

Building upon the reported findings, we postulated that linoleic acid could induce metabolic rewiring, thus restoring redox balance and mitigating the inflammatory response in UVB-irradiated keratinocytes. Our study substantiated this hypothesis through revealing significant metabolic alterations induced by linoleic acid, particularly affecting carbohydrate, amino acid, and glutathione metabolism. In conjunction with this metabolic reprogramming, linoleic acid was found to enhance the expression of critical enzymes involved in glutathione synthesis via the PI3K/Akt pathway while concurrently reducing the expression of COX-2 (cyclooxygenase-2). These findings offer preliminary insights into the mechanisms underlying linoleic acid’s antioxidant and anti-inflammatory effects during photoaging.

## 2. Results

### 2.1. Effect of LA on Cell Viability of HaCaT Keratinocyte Cells

**Figure 1:**
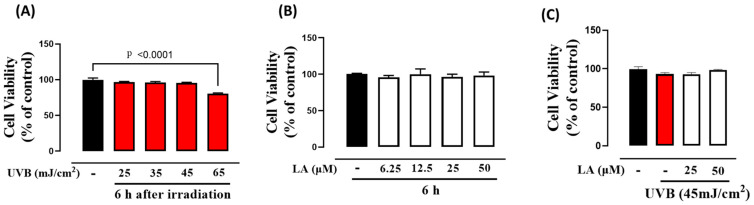
Determination of cell viability according to the CCK-8 (Cell Counting Kit-8) assay. The bar graphs represent the percentages of cell viability of HaCaT cells (**A**) irradiated with different doses of UVB, (**B**) treated with different concentrations of LA, and (**C**) irradiated with UVB and treated with 25 µM or 50 µM of LA. The evaluation of cell viability was determined at 6 h after treatment in all cases. Significance was calculated using the one-way ANOVA test and was corrected for multiple comparisons using Dunnett’s test. Black bars: non-irradiated cells; red bars: untreated UVB-irradiated cells; white bars: LA-treated cells.

In this study, we investigated the acute effects of different doses of UVB radiation. Specifically, we focused on assessing the short-term impact of UVB exposure, examining the effects at 6 h post-irradiation [6]. The survival rate of cells was evaluated by subjecting them to UVB irradiation at doses of 25, 35, 45, and 65 mJ/cm^2^, according to the dosing guidelines established by the American Academy of Dermatology and the dose range commonly employed in studies evaluating photodamage [22,23]. The survival rates of HaCaT cells at 6 h post-irradiation were 96.8%, 95.9%, 95.4%, and 80.6%, respectively (Figure 1A). A dose of 45 mJ/cm^2^ for subsequent experiments was selected, as it represented the highest UVB dose that did not significantly decrease cell viability at 6 h after irradiation. In addition, we observed no toxicity of LA at concentrations up to 50 µM after 6 h of incubation (Figure 1B). Moreover, when cells were exposed to 45 mJ/cm^2^ UVB radiation and subsequently treated with 25 µM or 50 µM LA for 6 h, there were no alterations in cell survival rates when compared to untreated irradiated cells (Figure 1C). However, given that UVB exposure triggers a substantial increase in the production of ROS in HaCaT keratinocytes, even at low doses [24], we proceeded to evaluate ROS production in HaCaT cells at 6 h after irradiation with 45 mJ/cm^2^ and subsequent treatment with 50 µM LA.

### 2.2. Effect of LA on Intracellular ROS Production in UVB-Irradiated Keratinocytes

**Figure 2:**
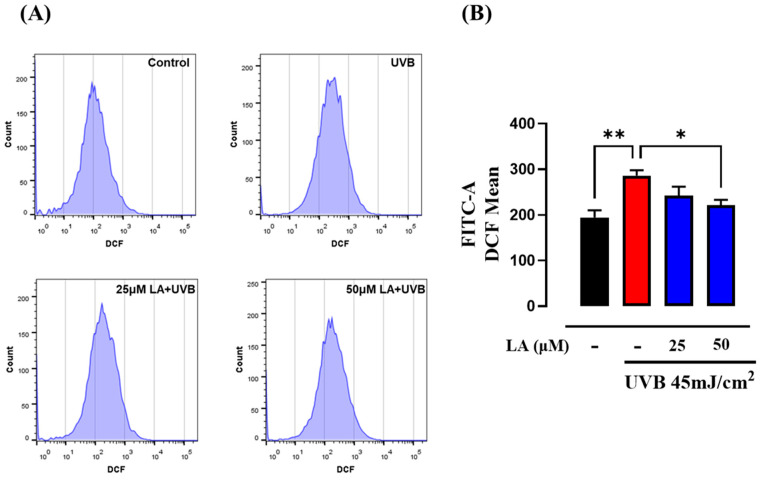
Effect of LA on UVB-induced ROS production in keratinocytes. (**A**) A representative histogram of four different experiments. A shift to the left of the histogram corresponding to the HaCaT cells irradiated and treated with 50 µM of LA can be observed, similar to that obtained from the non-irradiated control. (**B**) The average fluorescence intensity was analyzed using FACS. The average dichlorofluorescein (DCF) levels in the UVB-irradiated HaCaT cells were higher than in the non-irradiated control, while the average DCF values in the irradiated cells treated with 50 μM LA were lower than in the irradiated cells. Bar graphs represent the mean ± the standard error of the mean (SEM) from four independent experiments. * *p* < 0.05; ** *p* < 0.01 compared to cells exposed to UVB radiation without treatment. Black bar: non-irradiated cells; red bar: untreated UVB-irradiated cells; blue bars: UVB and LA-treated cells.

Previous research has shown that exposure to UVB radiation in the 30–100 mJ/cm^2^ range increases ROS production at 3 min in HaCaT human keratinocyte cell lines [25]. On the other hand, it has also been shown that UVB-induced ROS production remains high even 24 h after irradiation [26]. In this study, we observed that cellular fluorescence intensity, which indicates intracellular ROS levels, was increased at 6 h after the cells were exposed to 45 mJ/cm^2^ of UVB compared to non-irradiated cells (Figure 2). Furthermore, we evaluated the effects of 25 and 50 µM LA treatments on UVB-induced ROS production. Figure 2A presents a quantitative analysis of cellular ROS levels, while the bar graph in Figure 2B illustrates the fluorescence intensity derived from the flow cytometry results. We observed that treatment with 50 µM LA significantly decreased ROS production, compared to the UVB-irradiated control. Hence, these results indicate that, under these conditions, exposure to UVB radiation induces an increase in the production of ROS while treatment with linoleic acid reduces these levels.

It has long been recognized that energy metabolism is intricately linked to the generation of ROS, and critical enzymes associated with metabolic pathways can be influenced by redox reactions [27]. Nevertheless, the impact of UVB radiation exposure on keratinocyte metabolism remains relatively unexplored.

### 2.3. Differences in Metabolites of HaCaT Cells Exposed to UVB and Treated with Linoleic Acid

Previous research has shown that exposure to UVB radiation can produce metabolic reprogramming in a manner dependent on ROS production. For this reason, we evaluated the metabolic impact of a dose of 45 mJ/cm^2^ of UVB at 6 h post-irradiation in HaCaT cells. Using multivariate analysis, we aimed to characterize the metabolic alterations induced by UVB exposure and the effects of linoleic acid treatment.

**Figure 3:**
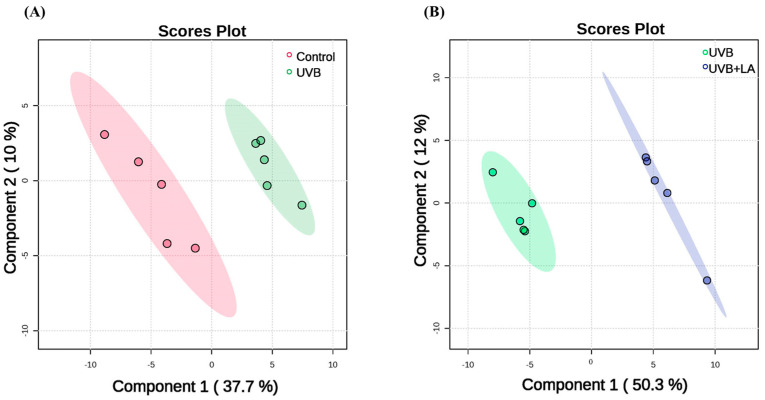
Partial least squares-discriminant analysis (PLS-DA) score plots based on metabolomic analysis. (**A**) HaCaT cells irradiated with 45 mJ/cm^2^ (green) and non-irradiated control cells (red) at 6 h post-irradiation. (**B**) Cells irradiated with 45 mJ/cm^2^ of UVB (green) and cells irradiated with UVB and treated with 50 µM LA (blue), at 6 h post-irradiation. The explained variances of the selected components are shown in brackets. *n* = 5, independent experiments.

Utilizing partial least squares-discriminant analysis (PLS-DA), we found that metabolite abundances alone effectively discriminated between samples, explaining 47.7% of the variation in the data through the first two principal components (PC1 and PC2) between control and UVB-irradiated cells (Figure 3A). Furthermore, when comparing UVB-irradiated cells with those treated with 50 µM LA, 62.3% of the variation was explained by the first two principal components (PC1 and PC2) (Figure 3B). These results suggest that the metabolite profiles of HaCaT cells irradiated with UVB were altered with respect to the non-irradiated control group, and the metabolic pattern altered by UVB was modified through treatment with LA.

**Figure 4:**
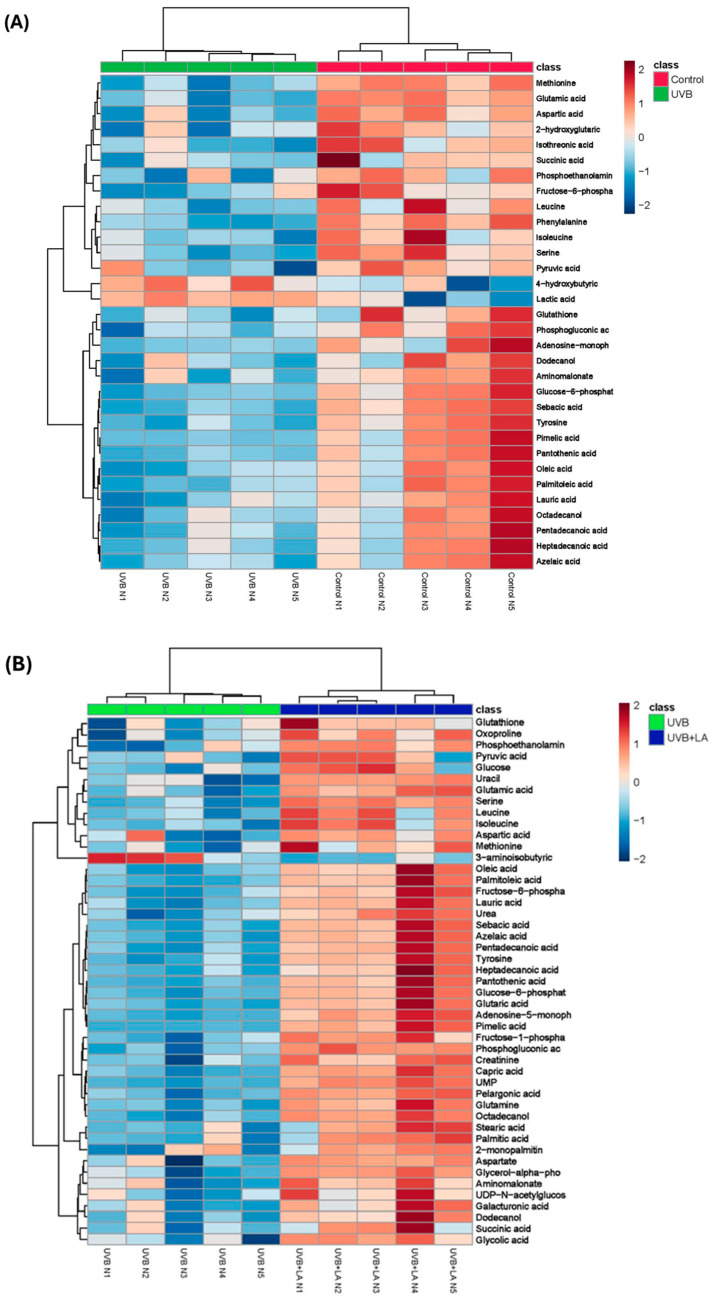
Heatmap of changes in metabolite profiles induced by (**A**) UVB radiation and (**B**) LA treatment in UVB-irradiated cells. Rows represent specific intracellular metabolites and columns represent conditions. *n* = 5, independent experiments.

To assess the metabolic alterations induced by UVB radiation and subsequent treatment with LA, we generated a heatmap incorporating 32 metabolites for the control vs. UVB and 47 metabolites for UVB vs. UVB + LA comparisons, selected based on the lowest *p*-values (Figure 4). The chemical classifications of metabolites in both groups predominantly included amino acids, fatty acids, carbohydrates, alpha-keto acids, secondary alcohols, phosphoethanolamines, and alpha hydroxy acids (Table 1 and Table 2).

Our observations revealed that UVB radiation reduced various amino acids, such as phenylalanine, glutamic acid, methionine, tyrosine, serine, aspartic acid, leucine, and isoleucine. Additionally, a decrease in the glutathione level was noted in the irradiated group. Moreover, there was a decline in critical fatty acids which are essential for skin maintenance, including sebacic acid, pimelic acid, palmitoleic acid, oleic acid, lauric acid, and azelaic acid. Intermediate metabolites associated with the TCA cycle and glycolysis, such as glucose-6-phosphate, succinic acid, and pyruvic acid, also exhibited decreased levels in cells exposed to UVB radiation. Conversely, lactic acid levels were increased in irradiated cells (Figure 4A). In Table 1, we present the fold changes (FCs), expressed as the ratios between the control group (non-irradiated cells) and the UVB-irradiated cells. The table highlights the metabolites that exhibited statistically significant changes between these two groups.

Subsequently, we investigated whether metabolic reprogramming occurred during LA treatment in response to UVB exposure. To this end, cells irradiated with 45 mJ/cm^2^ UVB were immediately treated with LA for 6 h. In contrast to observations in UVB-irradiated cells, LA treatment resulted in increased levels of specific amino acids, such as serine, glutamine, tyrosine, glutamic acid, isoleucine, leucine, aspartate, oxoproline, methionine, and aspartic acid. Similarly, LA treatment led to elevated levels of fatty acids that had decreased with UVB exposure, including capric acid, pelargonic acid, pimelic acid, azelaic acid, sebacic acid, pentadecanoic acid, lauric acid, palmitoleic acid, oleic acid, heptadecanoic acid, palmitic acid, and stearic acid. Furthermore, intermediate metabolites of the Krebs cycle and glycolysis, such as glucose-6-phosphate, succinic acid, and pyruvic acid, were restored upon LA treatment. Additionally, LA treatment reinstated glutathione levels, which decreased upon UVB exposure (Figure 4B). In Table 2, we present the FCs, expressed as the ratios of UVB + LA/UVB, of the metabolites that exhibited statistically significant changes between both groups.

### 2.4. Linoleic Acid Modifies Intracellular Metabolic Pathways in UVB-Irradiated Keratinocytes

**Figure 5:**
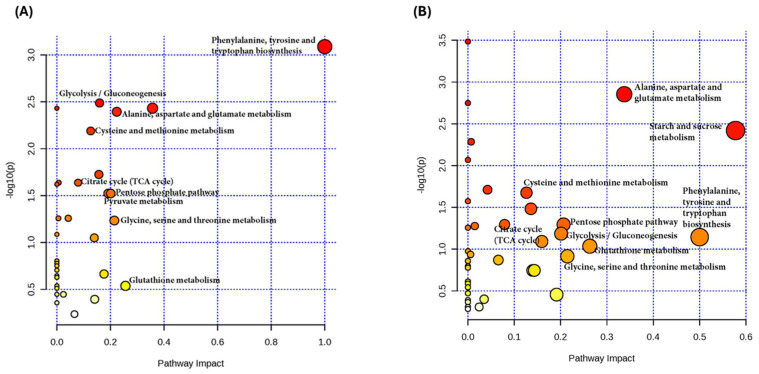
Overview of the main metabolic pathways affected by (**A**) radiation with 45 mJ/cm^2^ UVB and (**B**) HaCaT cells irradiated with UVB and treated with 50 µM LA, after 6 h. *n* = 5, independent experiments.

Significant metabolite differences between groups were identified using the MetaboAnalyst 6.0 software for metabolomic pathway analysis. The metabolic pathway impact diagram shows the metabolic pathways affected by the experimental conditions. The Y-axis (−log_10_(p)) indicates the statistical significance of each pathway, with higher values representing greater significance, and the X-axis (pathway impact) measures the impact of each pathway, with higher values indicating greater biological relevance. The size of the circles represents the impact of the metabolic pathway, with larger circles showing greater impact. The color of the circles indicates statistical significance, with redder circles representing higher significance (lower *p*-value). Based on this, the most affected metabolic pathways, both in terms of impact and significance, between the control and UVB-irradiated groups, encompass phenylalanine, tyrosine, and tryptophan biosynthesis; glycolysis/gluconeogenesis; cysteine and methionine metabolism; the TCA cycle; the pentose phosphate pathway; pyruvate metabolism; and glutathione metabolism (Figure 5A). In addition, metabolic pathway analysis indicated that the alterations in metabolites following linoleic acid treatment in UVB-exposed HaCaT cells primarily involved alanine, aspartate, and glutamate metabolism; starch and sucrose metabolism; cysteine and methionine metabolism; the pentose phosphate pathway; glycolysis/gluconeogenesis; the TCA cycle; and glutathione metabolism (Figure 5B).

### 2.5. Linoleic Acid Restores Glutathione Levels Reduced by UVB Exposure through the PI3K/Akt Pathway

**Figure 6:**
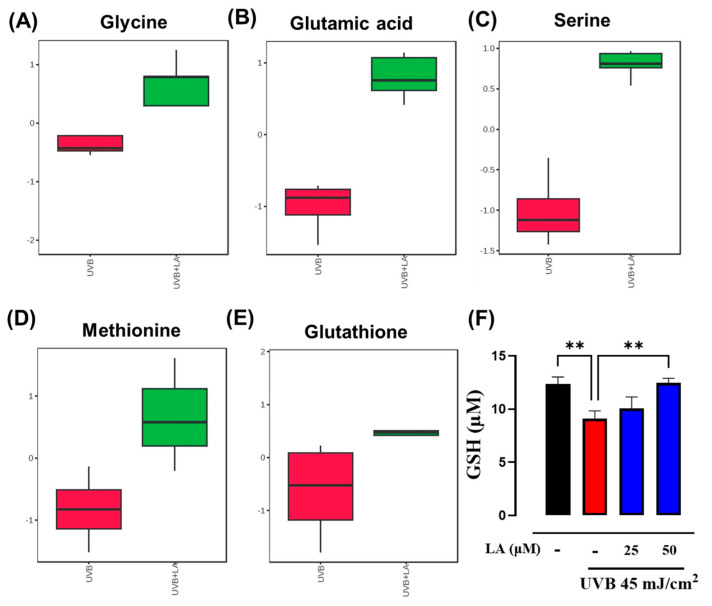
Effects of LA treatment on the synthesis of glutathione and its precursors in UVB-irradiated HaCaT cells. (**A**–**E**) Relative abundances of glutathione and typical metabolites related to their synthesis (axis Y = relative abundance with respect to ribitol). (**F**) Quantification of GSH with the method based on Ellman’s reagent (DTNB, 5,5′-dithio-bis-2-nitrobenzoic acid). Data are presented as mean ± SEM. ** *p* < 0.01, compared to control cells. *n* = 5, independent experiments. Black bar: non-irradiated cells; red bar: untreated UVB-irradiated cells; blue bars: UVB and LA-treated cells.

Given the crucial roles of oxidative stress and GSH metabolism in the skin following UVB exposure, our focus centered on modulating this metabolic pathway. Metabolic pathway analysis unveiled alterations in the metabolism of GSH precursors, including glutamate metabolism, cysteine and methionine metabolism, and glycine and serine metabolism (Figure 5). Furthermore, our study revealed that UVB irradiation led to a notable reduction in both the abundance of GSH precursors (cysteine, glutamate, and glycine) and GSH itself in cells. At the same time, LA treatment effectively increased these metabolites (Figure 6A–E). Subsequently, we quantified the concentration of GSH in cells irradiated and treated with LA. Consistent with the metabolomics data, the GSH concentration decreased significantly (by almost 30%) in irradiated cells compared to non-irradiated cells. Notably, treatment with LA restored GSH concentrations to basal levels (Figure 6F).

**Figure 7:**
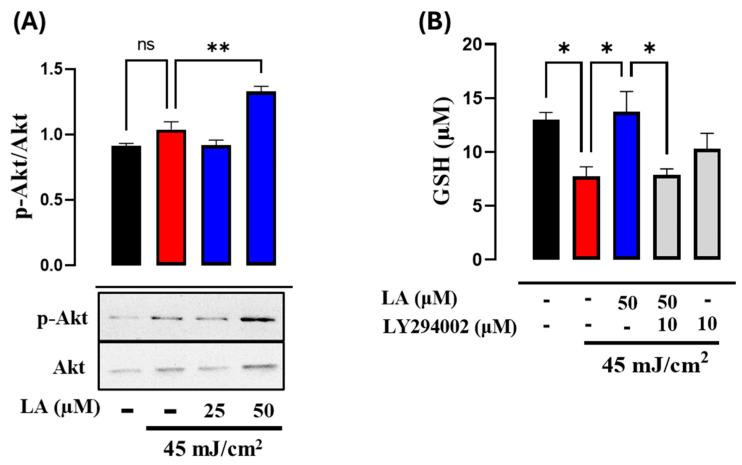
Involvement of the PI3K/Akt pathway in LA-induced GSH levels in UVB-irradiated HaCaT cells. (**A**) Levels of the phosphorylated form of Akt were analyzed by Western blot and total Akt was used as a loading control. A representative image from an experiment is shown (below). The densitometric ratios of p-Akt/Akt are shown in the bar graph as mean ± SEM (top). (**B**) Quantification of GSH in the presence or absence of the PI3K inhibitor, LY204002. * *p* < 0.05, ** *p* < 0.01, compared to the UVB control. *n* = 5, independent experiments. ns: not significant. Black bar: non-irradiated cells; red bar: untreated UVB-irradiated cells; blue bars: UVB and LA-treated cells; grey bars: cells treated with inhibitor LY294002.

A cellular regulatory response to UVB radiation stress is an increase in the PI3K/Akt pathway to promote GSH synthesis. To assess the effect of LA on the PI3K pathway, we measured the phosphorylation levels of its downstream effector, Akt, through Western blot analysis. p-Akt levels were elevated by UVB irradiation, compared to those in the control group, while LA further augmented p-Akt levels in UVB-irradiated cells (Figure 7A). To validate that the effect of linoleic acid on increasing GSH concentration was mediated through the PI3K/Akt pathway, cells were treated with a specific PI3K inhibitor, LY294002. Compared with the UVB group, the GSH concentration was increased in the UVB + LA group; however, after PI3K inhibition, LA-induced GSH levels decreased (Figure 7B). These findings suggest that LA exerts a protective effect through enhancing GSH levels by activating the PI3K-Akt pathway.

### 2.6. Effect of LA Treatment on GCLC and GSS Expression in UVB-Irradiated HaCaT Cells

**Figure 8:**
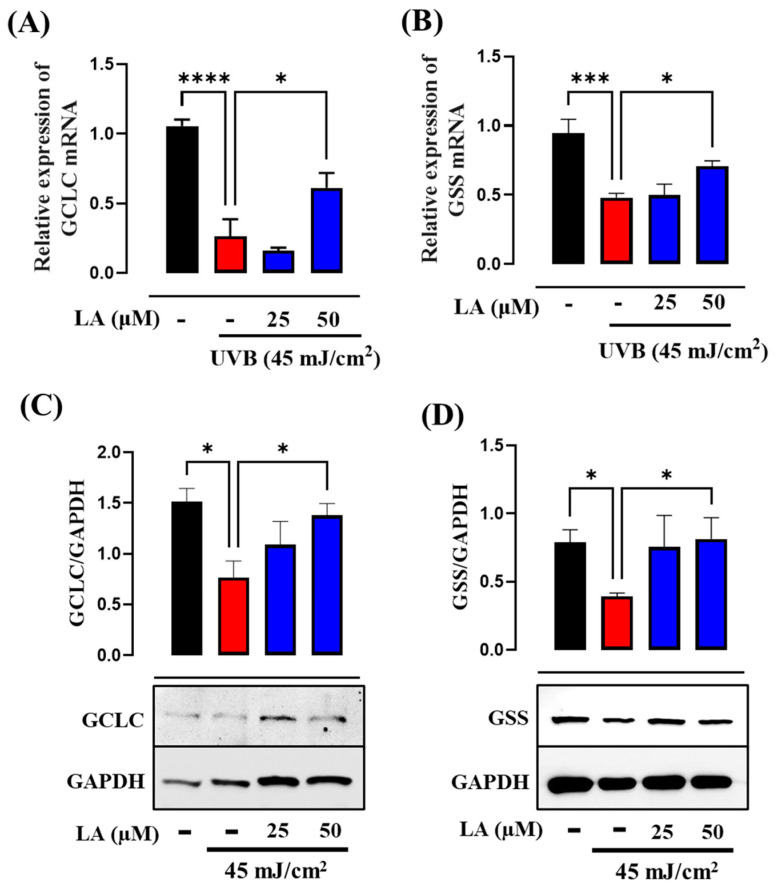
Linoleic acid increases GCLC and GSS levels in UVB-irradiated HaCaT cells. Bar graphs show the relative mRNA expression levels of (**A**) GCLC and (**B**) GSS. RPS9 was used as a housekeeping gene. The levels of the proteins (**C**) GCLC and (**D**) GSS were determined through Western blot and GAPDH was used as a loading control. Each bar represents the mean ± SEM. * *p* < 0.05; *** *p* < 0.005 and **** *p* < 0.001, compared to the UVB control. *n* = 5, independent experiments. Black bar: non-irradiated cells; red bar: untreated UVB-irradiated cells; blue bars: UVB and LA-treated cells.

**Figure 9:**
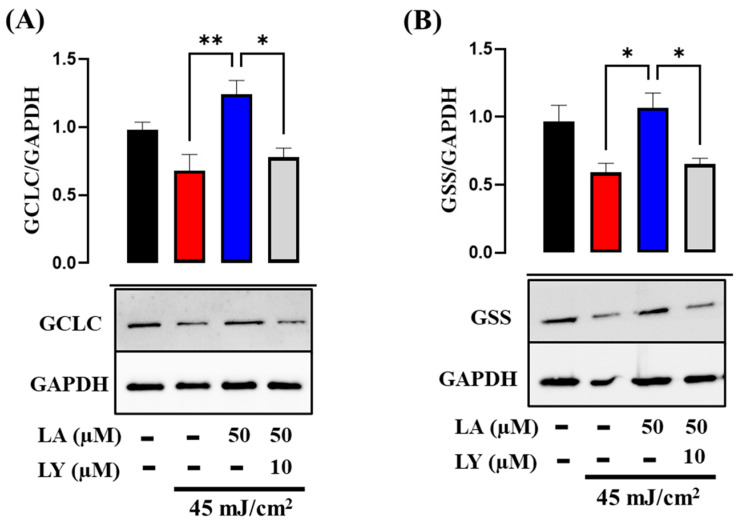
LA-induced GCLC and GSS expression is mediated through the PI3K/Akt pathway. Total proteins were analyzed through immunoblotting using specific antibodies against (**A**) GCLC and (**B**) GSS in the presence or absence of the PI3K inhibitor. GAPDH was used as a loading control. (Bottom) Images from a representative experiment are shown. (Top) Densitometric ratios of GCLC/GAPDH or GSS/GAPDH are shown in the graphs as mean ± SEM. * *p* < 0.05; ** *p* < 0.01 compared to the UVB control. *n* = 5, independent experiments. Black bar: non-irradiated cells; red bar: untreated UVB-irradiated cells; blue bar: UVB and LA-treated cells; grey bar: cells treated with inhibitor LY294002.

The PI3K–Akt–Nrf2 signaling axis serves as a principal determinant in the induction of GSH synthesis through the transcriptional activation of GCLC and GSS [28,29]. To determine the role of linoleic acid in GSH synthesis, we assessed the expression levels of GCLC and GSS. In accordance with other authors, we observed a decrease in the expression and synthesis of GCLC and GSS after exposure to UVB. On the other hand, treatment with 50 µM LA restored the levels of both enzymes (Figure 8A–D). These results suggest that the increase in GSH induced by LA is related to activation of the expression of enzymes critical for the de novo synthesis of GSH. Moreover, the PI3K inhibitor reduced the LA-induced levels of both proteins (Figure 9A,B). These results suggest that the expression of GCLC and GSS induced by LA is mediated through activation of the PI3K/Akt pathway.

### 2.7. LA Decreases UVB Radiation-Induced COX-2 Expression and PGE2 Synthesis

**Figure 10:**
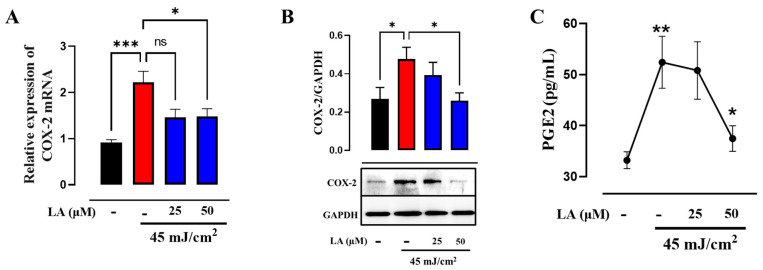
Linoleic acid reduces COX-2 expression and PGE2 synthesis in UVB-irradiated HaCaT cells. (**A**) Bar graphs show the relative mRNA expression levels of COX-2. RPS9 was used as a housekeeping gene. (**B**) The levels of proteins of COX-2 were determined through Western blot and GAPDH was used as a loading control. (**C**) The graph shows the concentration of PGE2 determined through ELISA. Each bar represents the mean ± SEM. * *p* < 0.05; ** *p* < 0.01; *** *p* < 0.005, compared to the UVB control. *n* = 5, independent experiments. ns: not significant. Black bar: non-irradiated cells; red bar: untreated UVB-irradiated cells; blue bars: UVB and LA-treated cells.

A well-established cross-talk exists between oxidative stress and inflammation [30]. Evidence suggests that the reduction in ROS by GSH inhibits NF-κB activation, thereby effectively regulating the cellular pro-inflammatory response [12]. NF-κB regulates the expression of COX-2, contributing to photoaging [31]. We evaluated COX-2 expression and found that UVB irradiation in HaCaT cells increased COX-2 expression and elevated prostaglandin E2 (PGE2) levels. However, treatment with LA suppressed UVB-induced COX-2 and PGE2 expression (Figure 10A–C). These findings confirm that linoleic acid suppresses UVB-induced pro-inflammatory mediators in HaCaT cells.

## 3. Discussion

Acute and chronic exposure of the skin to UV radiation triggers the production of ROS, DNA damage, and inflammation, leading to accelerated aging and the potential for development of skin cancer. Omega-6 polyunsaturated fatty acids (PUFAs), such as linoleic acid, play essential roles in numerous metabolic pathways and have been shown to possess antioxidant properties. However, the precise mechanisms by which omega-6 PUFAs mitigate UVB-induced oxidative stress in the skin remain incompletely understood. In this study, we investigated the effects and underlying mechanisms of linoleic acid—an omega-6 PUFA—on keratinocytes following acute UVB radiation exposure.

Our results revealed that UVB radiation significantly elevated intracellular ROS production and perturbed the metabolism of amino acids, fatty acids, and various carbon pathways, including glutathione metabolism. However, linoleic acid treatment effectively mitigated UVB-induced ROS levels and restored the metabolic pathways disturbed by UVB radiation. Mechanistically, linoleic acid exerted its protective effects by enhancing GSH levels through upregulation of GCLC and GSS expression via the PI3K/Akt signaling pathway. Moreover, linoleic acid effectively dampened the expression levels of COX-2 and PGE2. These findings highlight the effect of linoleic acid as a protective agent against UVB-induced skin damage, providing valuable insights into its mechanisms of action in modulating oxidative stress and inflammation.

Numerous investigations have utilized the HaCaT cell line to explore the protective or reparative effects of chemical compounds against UVB radiation-induced damage. However, the cellular response varies with the dose of UV irradiation, as demonstrated by Mammon et al. [32]. Moreover, the post-irradiation time is crucial to consider when evaluating these effects. Given that UVB-induced damage plays a key role in the development of other skin pathologies, our goal is to investigate LA’s ability to reverse or reduce such UVB-induced damage; hence, LA treatments were done after UVB irradiation.

HaCaT cells are a well-established and widely characterized model for the study of human keratinocytes, having been used extensively in research on UVB-induced damage, oxidative stress, and inflammatory responses [33,34]. Their stability, robustness, and reproducibility make them especially suitable for experiments seeking to assess general mechanisms of cellular damage and protection mechanisms, without the intrinsic variability presented by primary keratinocytes. Although the p53 mutation present in HaCaTs could be a limitation for studies focused exclusively on DNA damage repair, this fact also makes them a relevant model to investigate pathological conditions where p53 is altered, such as actinic keratoses and squamous cell carcinomas [35]. These mutations are frequent in tissues exposed to UV radiation, which reinforces the usefulness of HaCaT cells for studies on photocarcinogenesis and chronic sun damage. Hence, HaCaT cells are suitable for preliminary studies exploring general cellular responses to oxidative stress and UVB damage. However, for more specific experiments on DNA repair, the use of primary keratinocytes may be more appropriate, providing complementary results that offer more detailed and physiologically relevant insight. 

Our study evaluated a dose range of 15–65 mJ/cm^2^ and assessed the effects in the short-term post-irradiation period of 6 h [6]. We observed that increasing UVB doses led to significant toxicity in HaCaT cells. Consequently, we selected the highest dose that did not show apparent cytotoxicity: 45 mJ/cm^2^. Despite the absence of cytotoxic effects, we found that exposure to 45 mJ/cm^2^ of UVB increased ROS levels, while treatment with 50 μM linoleic acid for 6 h effectively decreased UVB-induced ROS. Previous research on human keratinocytes has also demonstrated a dose-dependent increase in ROS production following UVB radiation in a dose range of 1–100 mJ/cm^2^ [2].

UVB radiation stands as a primary contributor to skin cancer [36], with documented evidence showcasing its ability to induce metabolic changes in both keratinocytes and fibroblasts [8,36]. Furthermore, in a multi-stage murine model of UVB radiation-induced skin cancer, it was revealed that glycolysis, the TCA cycle, and fatty acid β-oxidation are diminished at an early stage of photocarcinogenesis. This study underscored how UVB radiation disrupts various metabolic pathways within 6 h post-irradiation.

Our research corroborated these findings through demonstrating that UVB radiation inhibits amino acid metabolism and diminishes carbon metabolism pathways, including glycolysis/gluconeogenesis, the citrate cycle, the pentose phosphate pathway, and glutathione metabolism. Amino acids, serving as fundamental constituents of proteins, are intricately involved in diverse cellular processes, including energy generation. Our investigation highlighted the significant reduction in several amino acids within keratinocytes induced by UVB radiation, indicative of altered cellular metabolism.

Furthermore, consistent with findings from other studies, UVB radiation markedly hindered glutathione metabolism and disrupted the metabolism of glutathione precursor amino acids. Glutathione, which is synthesized from cysteine, glutamate, and glycine, is pivotal in maintaining the cell’s antioxidant defense systems. UVB radiation has been shown to impede glutathione synthesis, leading to oxidative stress induction and promoting keratinocyte apoptosis [37].

Glutathione is a crucial intracellular mediator for cellular defense against reactive oxygen intermediates. Apart from acting as a substrate for glutathione-dependent antioxidant enzymes, this thiol tripeptide facilitates the regeneration of ascorbate and α-tocopherol. Moreover, it directly detoxifies reactive species through conjugating pro-oxidants [38,39]. Other experiments have revealed that depleting keratinocytes of glutathione using buthionine sulfoximine—an inhibitor of glutathione synthesis—led to a significant increase in intracellular levels of ROS [2]. Furthermore, glutathione-depleted cells exhibited heightened sensitivity to the oxidant-generating effects of UVB light.

These findings underscore the critical role of glutathione in mitigating the accumulation of potentially harmful reactive oxygen species in UVB-treated keratinocytes. These insights align with our results, emphasizing the significance of glutathione metabolism in safeguarding against photodamage. Given the pivotal roles of glutathione synthesis and oxidative stress in conditions like psoriasis, we further delved into this pathway.

Our observations revealed that treatment of UVB-irradiated cells with linoleic acid effectively reversed the metabolic alterations induced by UVB. In particular, LA supplementation increased intracellular levels of amino acids, free fatty acids, and glutathione, restoring the metabolic pathway disruptions caused by UVB radiation. These outcomes suggest that linoleic acid can induce metabolic reprogramming in cells perturbed by UVB exposure. Notably, a prominent feature was the augmentation of GSH and its precursor amino acids in cells treated with LA. Hence, we can suggest that the increase in GSH levels induced by LA could be related to restoring the energy metabolism altered by UVB radiation. The relationship between GSH and the restoration of energy metabolism altered by UVB radiation is closely linked to the crucial role of GSH in maintaining cellular redox balance and protecting mitochondria from oxidative stress. When cells are exposed to UVB radiation, an increase in the production of ROS is generated, which can compromise mitochondrial function and alter energy metabolism. GSH, as a key antioxidant, neutralizes ROS and promotes the repair of oxidative damage, facilitating the restoration of normal mitochondrial function and energy production.

While numerous studies have highlighted the antioxidant effects of vegetable oils rich in linoleic acid, the cellular mechanisms underlying the antioxidant properties mediated by LA remain relatively obscure [20]. However, recent research has demonstrated that omega-6 polyunsaturated fatty acids, including LA, exert antioxidant effects through the Keap1–Nrf2 system in hepatocytes, although the exact mechanisms and signaling pathways involved remain incompletely understood [21]. We demonstrate that linoleic acid regulates the de novo synthesis of GSH by increasing the enzymes involved in its production, which are transcriptionally regulated by the nuclear factor erythroid 2-related factor 2 (Nrf2) pathway.

Inducing antioxidant genes could be an effective strategy for safeguarding against oxidation caused by environmental stressors. In our study, we observed that LA treatment heightened the expression of GCLC and GSS in UVB-exposed keratinocytes.

GSH is biosynthesized de novo through the successive action of two enzymes that conjugate L-glutamine, L-cysteine, and glycine. The initial enzyme in this process is glutamate–cysteine ligase (GCL), also known as γ-glutamylcysteine synthase, which synthesizes γ-glutamylcysteine. Subsequently, GSH synthetase (GSS) catalyzes the linkage of glycine with γ-glutamylcysteine to form GSH. It is widely accepted that GCLC, rather than GSS, serves as the rate-limiting enzyme in GSH biosynthesis [40]. Moreover, both enzymes are transcriptionally regulated by the Keap1–Nrf2 pathway.

Given GSH’s pivotal role in cellular defense mechanisms, the induction of regulatory enzymes involved in its synthesis is a crucial defense mechanism.

Using the PI3K inhibitor LY294002, we demonstrated that inhibition of the PI3K/Akt pathway resulted in a reduction in LA-induced GSH levels in UVB-irradiated keratinocytes. LY294002 blocks the activity of PI3K through direct interaction with the p85 regulatory subunit. At concentrations inhibitory to Akt phosphorylation, LY294002 also suppressed basal GSH synthesis. Thus, based on our findings, a functional PI3K/Akt pathway is necessary for the LA-mediated augmentation of GSH levels.

The expression of antioxidant genes downstream of Nrf2 can display coordinated or discordant responses depending on the treatment conditions and the genes assessed. Nrf2 contains five ECH homology domains which interact with various transcription factors and cofactors, imparting specificities towards different promoters [41].

UVB-induced ROS production activates MAPK signaling and the transcription factor NF-κB, leading to inflammation. It has been proposed that by mitigating ROS production, GSH inhibits NF-κB activation, thereby regulating the cytokine storm [42]. Furthermore, other researchers have shown that NF-κB undergoes modification through S-glutathionylation, a process involving the conjugation of GSH to cysteines via mechanisms that are not yet fully understood [43]. S-glutathionylation of IKKβ inhibits its kinase activity and downstream pro-inflammatory responses to lipopolysaccharide (LPS) [44]. Additionally, S-glutathionylation prevents the ubiquitination and subsequent degradation of IκBα, as well as the DNA binding of RelA/p50 dimers, making it a crucial mechanism in the regulation of NF-κB activation [43]. NF-κB regulates COX-2, and we observed an increase in COX-2 expression and its product PGE2 in UVB-irradiated keratinocytes, while treatment with LA reduced both COX-2 and PGE2 levels. Prostaglandins generated by the arachidonic acid cascade are involved in tumorigenesis, and elevated levels of PGE2 in skin carcinomas are associated with increased metastatic and invasive behavior, highlighting the importance of controlling the increase induced by UVB radiation [45].

We previously reported that LA has a pro-inflammatory effect in normal keratinocytes, primarily mediated through the free fatty acid receptor 1 (FFAR1) [46]. However, in this study, we observed the opposite effect: LA acted as an anti-inflammatory agent in cells exposed to UVB radiation. This aligns with studies showing that LA’s effects can vary significantly depending on the inflammatory context versus basal conditions, highlighting its protective role in skin inflammation models [46,47,48,49]. This duality suggests that LA’s impact on the skin depends on the cellular environment and physiological state. It appears to function as a pro-inflammatory agent under normal conditions, while exerting anti-inflammatory effects in the presence of oxidative stress or UVB-induced inflammation. This differential behavior underscores the complexity of LA’s role in skin biology.

The results of this work underscore the significant effects of linoleic acid on UVB-induced damage in HaCaT cells, offering valuable insights into how linoleic acid interacts with the epidermis to mitigate this damage. The relevance of these findings for human keratinocytes, combined with the controlled experimental environment, enables a deeper investigation into its antioxidant, anti-inflammatory effects, and its role in metabolic reprogramming. Moreover, this study is the first to identify the mechanisms through which linoleic acid mitigates UVB-induced cellular damage. This approach serves as a powerful tool in the early stages of research, laying the groundwork for future studies in critical areas such as skin health, photoaging, and skin cancer prevention.

These findings offer preliminary insight into the underlying mechanisms of linoleic acid’s antioxidant and anti-inflammatory properties in the photoaging process, suggesting that linoleic acid may play a crucial role in cellular protection against oxidative damage and inflammation caused by UV radiation exposure.

## 4. Materials and Methods

### 4.1. Cell Culture

The immortalized human keratinocytes, HaCaT cells [50], were generously provided by Dr. Miguel Concha at the Department of Pathology, Faculty of Medicine, Universidad Austral de Chile, Valdivia, Chile. The cells were cultured in Dulbecco’s modified Eagle’s medium (DMEM; HyClone, GE Healthcare Life Sciences, Logan, UT, USA) supplemented with 10% fetal bovine serum (FBS; Gibco, Grand Island, NY, USA) and antibiotics [100 U/mL of penicillin and 100 µg/mL streptomycin (Invitrogen, Thermo Fisher Scientific, Waltham, MA, USA)] at 37 °C in a humidified incubator with 5% CO_2_. HaCaT cells were grown until they reached 90% confluency and were then subcultured onto corresponding plates for assays. Before all experiments, the cells were incubated in serum-free DMEM medium for 24 h.

### 4.2. UVB Irradiation

For UVB irradiation, HaCaT cells were seeded in 6-well plates (3 × 10^5^ cells/well), 12-well plates (1 × 10^5^ cells/well), and 96-well plates (1 × 10^4^ cells/well) and cultured until reaching 80% confluence over 24 h. Before irradiation, cells were rinsed twice with phosphate-buffered saline solution (PBS), leaving a thin solution layer to prevent cell surface drying. Irradiation was performed using a 3UV series UV hand lamp (UVP, LLC, Upland, CA, USA) emitting 302 nm radiation. Irradiation doses were calculated using the following formula: Dose (mJ/cm^2^) = Exposure time (s) × Intensity (mW/cm^2^) [51,52]. Linoleic acid treatments were administered immediately post-irradiation, and cells were incubated for six hours under the same conditions as described above. The stock solution of linoleic acid (100%) was stored at −20 °C, hermetically sealed and protected from light. To perform the experiments, LA was dissolved in dimethyl sulfoxide (DMSO) at a concentration of 354 mM. From this stock, working solutions were prepared in DMEM medium. LA was kept in amber tubes protected from light, and oxygen was displaced with an inert gas to retard the oxidation process.

### 4.3. Cell Viability Assay

Cell viability under different conditions was assessed using the cell counting kit-8 (#96992 CCK-8; Sigma-Aldrich, St. Louis, MO, USA) method. HaCaT cells were cultured in 96-well plates until confluency was reached and then incubated in serum-free medium for 24 h. Cell irradiation was carried out as described previously, utilizing doses of 25, 35, 45, and 65 mJ/cm^2^. To evaluate the cytotoxic effect of linoleic acid, cells were treated with concentrations of 6.25, 12.5, 25, and 50 µM. The range of concentrations evaluated in our study is grounded in previous research on the use of linoleic acid in cell cultures [21,53,54,55,56,57]. Additionally, cell viability was assessed in UVB-irradiated cells treated with linoleic acid. Following the treatments, cells were incubated for 6 h. Subsequently, 10 µL of CCK-8 solution was added to each well, and the plate was further incubated for 2 h at 37 °C in a humidified incubator with 5% CO_2_. Optical density was measured at 450 nm using a plate reader (Stat Fax^®^ 2100, Palm City, FL, USA).

### 4.4. Intracellular ROS Detection

The fluorescent probe 5-(and-6)-chloromethyl-2′,7′-dichlorodihydrofluorescein diacetate (CM-H2DCFDA; #C6827 Invitrogen, Carlsbad, CA, USA) was employed to assess intracellular ROS levels. Cells were cultured in 6-well plates and treated as described previously. Following a 6-h incubation period, the cells were stained with 20 μM CM-H2DCFDA for 30 min at 37 °C in a CO_2_ incubator. Subsequently, cells were harvested using trypsin, and the cell pellet was washed twice with PBS. Fluorescence was analyzed using flow cytometry (FACSCalibur™; Becton-Dickinson, San Jose, CA, USA), and data analysis was conducted with the FlowJo 7.6 software.

### 4.5. Preparation of Samples for Metabolomic Analysis via Gas Chromatography–Mass Spectrometry (GC-MS)

The cells were cultured in 6-well plates and subjected to treatments to extract metabolites. Metabolites were extracted via cell lysis using 1 mL of cold extraction buffer composed of 37.5% vol/vol acetonitrile, 37.5% *vol*/*vol* isopropanol, and 25% *vol*/*vol* water containing 1 mM ribitol (#A5502, Sigma-Aldrich, St. Louis, MO, USA) as an internal standard. Cells were scraped off the plates, collected, and centrifuged at 14,000× *g* for 2 min at 4 °C. The resulting supernatant containing the metabolites was dried using a SpeedVac concentrator (Savant R SPD131DDA, Thermo Fisher Scientific, Waltham, MA, USA) at 45 °C for 90 min under 10 atm pressure. Once dried, the samples were washed with a buffer containing 50% *vol*/*vol* acetonitrile and 50% *vol*/*vol* water, followed by centrifugation at 14,000× *g* for 2 min at 4 °C, and the supernatants were evaporated to dryness.

The dried samples underwent derivatization using 10 μL of methoxyamine/pyridine hydrochloride solution (20 mg/mL; #226904, Sigma-Aldrich, #107463, Merck KGaA, Darmstadt, Germany) at 30 °C for 90 min. Subsequently, 90 µL of the derivatizing agent N-ethyl-N-(trimethylsilyl)-trifluoroacetamide (MSTFA) was added, along with 1% trimethylchlorosilane (TMCS) (#TS-48915, Thermo Fisher Scientific, Pierce Biotechnology, Rockford, IL, USA). Retention time markers consisting of a mixture of FAME C8–C30 (#400505, Fiehn GC/MS Metabolomics Standards Kit, Agilent Technologies, Santa Clara, CA, USA) were utilized. Finally, the samples were transferred into glass vials (#5181-8872, Agilent Technologies) equipped with screw caps (#8010-0543, Agilent Technologies).

### 4.6. Metabolomics via GC-MS

Derivatized samples (2 µL) were introduced into an Agilent 7890B GC system, which was coupled to a 5977A mass selective detector system operating in electron impact ionization (EI) mode (Agilent Technologies, Palo Alto, CA, USA). This injection was facilitated using an Agilent 7693 series autosampler (Agilent Technologies, Palo Alto, CA, USA) onto a 30 m × 0.25 mm × 0.25 μm DB-5 column (Agilent Technologies, Palo Alto, CA, USA). The injector temperature was maintained at 250 °C, and helium was utilized as the carrier gas with a flow rate set to 1 mL/min, initially with an oven temperature of 60 °C. Subsequently, the oven temperature was ramped at 10 °C/min until reaching 325 °C, with a total runtime of 37.5 min. A solvent delay of 5.9 min was applied before full spectra (50–600 *m*/*z*; 1.7 scans/s) were acquired at a digital scan rate of 20 Hz. The MS ion source temperature was set at 250 °C, with a quadrupole temperature of 150 °C. Metabolite identification was conducted following the procedures outlined by Fiehn [58], with specific parameters as follows: a retention index tolerance of 2000, EI similarity cutoff of 65%, identification score cutoff of 70%, mass scale tolerance of 0.5 Da, and a retention time tolerance of 0.5 min.

### 4.7. Glutathione Measurements

Cells were cultured in 6-well plates for the determination of glutathione and treated as previously described. Upon treatment, the cells were harvested by scraping and prepared for the measurement of total intracellular glutathione (GSH + GSSG) utilizing a glutathione assay kit based on Ellman’s reagent (DTNB, 5,5′-dithio-bis-2-nitrobenzoic acid), following the manufacturer’s protocol (Cayman Chemical, #703002).

Briefly, in this method, GSH undergoes oxidation by the reagent DTNB (5,5’-dithio-bis (2-nitrobenzoic acid)), leading to formation of the yellow compound TNB (5’-thio-bis (2-nitrobenzoic acid) 2-nitrobenzoic). The production of TNB is directly proportional to the concentration of GSH present in the sample and is detectable at a wavelength of 412 nm. Calibration was performed using the GSSG standard in a concentration range of 0.25–8 µM to determine the total GSH values in the samples.

### 4.8. Western Blot Analysis

Cells were washed twice with PBS and then lysed in RIPA buffer supplemented with phosphatase and protease inhibitors (#A32963, Thermo Scientific, Waltham, MA, USA). The cell lysate was centrifuged for 20 min at 17,000× *g* at 4 °C to eliminate cell debris. Protein concentrations were determined using the Bradford method, and equivalent amounts of protein by weight were combined with Laemmli buffer, boiled for 5 min at 95 °C, separated on 10% polyacrylamide gels, and transferred onto nitrocellulose membranes. The membranes were blocked with 5% skim milk in TBS-T (20 mM Tris-HCl, pH 7.5; 137 mM NaCl; 0.1% Tween 20) for 2 h. Subsequently, the membranes were incubated overnight at 4 °C with primary antibodies (diluted 1:1000). The primary antibodies utilized were as follows: anti-pAKT monoclonal antibody (#sc-271966; Santa Cruz Biotechnology, Dallas, TX, USA), AKT (#sc-81434; Santa Cruz Biotechnology), GCLC (#sc-390811; Santa Cruz Biotechnology), GSS (#sc-166882; Santa Cruz Biotechnology), COX-2 (#sc-19999; Santa Cruz Biotechnology), and GAPDH (#sc-365062; Santa Cruz Biotechnology).

The following day, the membranes were incubated with HRP-conjugated anti-mouse IgG antibody (#115-035-003; Jackson Immunoresearch, West Grove, PA, USA). Specific bands were visualized utilizing an enhanced chemiluminescence system (UVP ChemiD-itTS2., Upland, CA, USA), and band density was quantified using the ImageJ 1.35s software.

### 4.9. Quantitative Real-Time RT-PCR (qRT-PCR)

Total RNA was extracted from the HaCaT cells using the E.Z.N.A. Total RNA kit I (#R6834-01, Omega Bio-Tek, Norcross, GA, USA). To eliminate genomic DNA contamination, the RNA underwent treatment with the Turbo DNase-Free kit (#AM1907, AmbionTM, Thermo Fisher Scientific, Waltham, MA, USA). Subsequently, 200 ng of RNA was reverse-transcribed for cDNA synthesis utilizing M-MLV reverse transcriptase (#M5313, Promega, Madison, WI, USA), following the manufacturer’s instructions.

RT-qPCR assays were conducted using Takyon Rox SYBR MasterMix (#UF-RSMT-B0701, Eurogentec, Seraing, Belgium) with the following primers: GSS F-5′GGAGGAAAGGCGAACTAGTGTT3′ and R-5′CAGCTCCTCTAGCTGCTGTTT3′; GCLC F-5′CACCCTCGCTTCAGTACCTT3′ and R-5′TGGTACATTGATGACAACCTTTTCT3′; COX-2 F-5′ACTGCTCAACACCGGAATTT′3 and R-5′TCCAAAATCCCTTGAAGTGGG3′; and RPS9 F-5′ACGCTTGATGAGAAGGACCC3′ and R-5′ATCTGCCCTCATCCAGCAC-3′. RT-qPCR was performed on a real-time PCR system, StepOne Plus (Applied Biosystems, Thermo Fisher Scientific, Waltham, MA, USA), with the following cycling conditions: 1 cycle at 95 °C for 10 min, followed by 40 cycles at 95 °C for 30 s, 60 °C for 30 s (annealing), and 72 °C for 30 s (extension). Changes in expression were determined using the 2^−(ΔΔCt)^ method, as described by Livak and Schmittgen [59]. For normalization, RPS9 was employed as a housekeeping gene.

### 4.10. PGE2 Quantification via Enzyme-Linked Immunoassay (ELISA)

The supernatants obtained from the qPCR assay were centrifuged at 600× *g* for 6 min and utilized to estimate the concentration of PGE2, employing a PGE2 ELISA kit (#KGE004B, R&D Systems, NE, Minneapolis, MN, USA), following the manufacturer’s instructions. The capture antibody was initially added to a 96-well plate and incubated at 37 °C for 45 min. After several washes, HRP-conjugated streptavidin solution was added and incubated for 30 min at 37 °C. Subsequently, TMB solution was added, and the plate was incubated in the dark for 15 min. The reaction was terminated with 0.16 M H_2_SO_4_, and the plate was read using spectrophotometry at 450 nm in a plate reader (Stat Fax^®^ 2100, Palm City, FL, USA). The data obtained were analyzed using the GraphPad Prism 8.0 software.

### 4.11. Statistical Analyses

The results were analyzed via a one-way analysis of variance (ANOVA) and Dunnett’s multiples comparison test. Tukey’s test was used to determine the significant differences between groups. The study used the GraphPad Prism v5.0 software (Graph Pad Software Inc., La Jolla, CA, USA). *p* < 0.05 was considered significant. To determine the number of samples for experiments, we used the G*Power 3.1 program. Based on an effect size of 3.15, with a power of 95% and a significance level of 0.05, the calculation suggests that approximately four samples per group would be necessary. For all experiments, we used *n* = 5; however, although this sample size meets the statistical requirements, a more significant number of samples could increase the precision in estimating the effects and improve the robustness of the results.

## Figures and Tables

**Table 1:** List of metabolites from UVB-irradiated HaCaT cells.

Compound Name	HMDB	KEGG	Class	*p* Value	Fold Change
Phenylalanine	HMDB0000159	C00079	Amino acids	5.53 × 10^−1^	31.934
Glutamic acid	HMDB0000148	C00302	Amino acids	0.0001061	71.012
Methionine	HMDB0000696	C00073	Amino acids	0.0002292	25.672
Tyrosine	HMDB0000158	C00082	Amino acids	0.00072693	19.628
Serine	HMDB0000187	C00065	Amino acids	0.0011633	75.856
Aspartic acid	HMDB0000191	C00049	Amino acids	0.0031473	48.696
Leucine	HMDB0000687	C00123	Amino acids	0.011216	26.843
Isoleucine	HMDB0000172	C00407	Amino acids	0.015096	24.501
Sebacic acid	HMDB0000792	C08277	Fatty acids	8.97 × 10^−1^	68.367
Pimelic acid	HMDB0000857	C02656	Fatty acids	0.002975	65.839
Palmitoleic acid	HMDB0003229	C08362	Fatty acids	0.0051333	34.674
Oleic acid	HMDB0000207	C00712	Fatty acids	0.0063521	37.281
Lauric acid	HMDB0000638	C02679	Fatty acids	0.0072525	36.156
Azelaic acid	HMDB0000784	C08261	Fatty acids	0.010646	49.394
Pentadecanoic acid	HMDB0000826	C16537	Fatty acids	0.016192	30.464
4-hydroxybutyric acid	HMDB0000710	C00989	Fatty acids	0.024935	0.16946
Dodecanol	HMDB0011626	C02277	Fatty acids	0.039514	32.804
Glucose-6-phosphate	HMDB0001401	C00092	Carbohydrates	0.0004312	15.232
Phosphogluconic acid	HMDB0001316	C00345	Carbohydrates	0.0059728	47.516
Fructose-6-phosphate	HMDB0000124	C00085	Carbohydrates	0.018713	23.651
Glutathione	HMDB0000125	C00051	Peptides	0.028095	84.197
Lactic acid	HMDB0000190	C00186	Alpha hydroxy acids	0.010135	0.077605
Aminomalonate	HMDB0001147	C00872	Carboxylic acids	0.012716	36.743
Succinic acid	HMDB0000254	C00042	Dicarboxylic acids	0.036359	21.655
Phosphoethanolamine	HMDB0000224	C00346	Phosphate esters	0.039367	26.559
Pyruvic acid	HMDB0000243	C00022	Alpha-keto acids	0.035405	4.146
2-hydroxyglutaric acid	HMDB0000606	C01087	Short-chain hydroxy acids	0.031068	32.487
Adenosine-5-monophosphate	HMDB0000045	C00020	Purine ribonucleotides	0.011568	23.637
Pantothenic acid	HMDB0000210	C00864	Secondary alcohols	0.0023396	51.309

Fold changes (FCs) described are the ratios of the control/UVB. The class, FC, and *p* value were obtained from MetaboAnalyst v6.0.

**Table 2:** List of metabolites from UVB-irradiated HaCaT cells treated with 50 µM LA.

Compound Name	HMDB	KEGG	Class	*p* Value	Fold Change
Serine	HMDB0000187	C00065	Amino acids	1.61 × 10^−1^	47.945
Glutamine	HMDB0000641	C00064	Amino acids	0.00011024	39.576
Tyrosine	HMDB0000158	C00082	Amino acids	0.00034047	34.679
Glutamic acid	HMDB0000148	C00302	Amino acids	0.0004259	33.707
Isoleucine	HMDB0000172	C00407	Amino acids	0.0016414	27.848
Leucine	HMDB0000687	C00123	Amino acids	0.002599	25.852
Aspartate	HMDB0006483	C00402	Amino acids	0.0036844	24.336
Oxoproline	HMDB0000267	C01879	Amino acids	0.0081244	20.902
Methionine	HMDB0000696	C00073	Amino acids	0.015337	18.143
Aspartic acid	HMDB0000191	C00049	Amino acids	0.04182	13.786
Capric acid	HMDB0000511	C01571	Fatty acids	1.38 × 10^−1^	48.591
Pelargonic acid	HMDB0000847	C01601	Fatty acids	2.84 × 10^−1^	45.469
Pimelic acid	HMDB0000857	C02656	Fatty acids	3.57 × 10^−1^	44.469
Azelaic acid	HMDB0000784	C08261	Fatty acids	0.00018891	37.237
Sebacic acid	HMDB0000792	C08277	Fatty acids	0.00020543	36.873
Pentadecanoic acid	HMDB0000826	C16537	Fatty acids	0.00032321	34.905
Lauric acid	HMDB0000638	C02679	Fatty acids	0.00035312	34.521
Palmitoleic acid	HMDB0003229	C08362	Fatty acids	0.00036091	34.426
Oleic acid	HMDB0000207	C00712	Fatty acids	0.000997	30.013
Heptadecanoic acid	HMDB0002259	C06424	Fatty acids	0.00272	25.654
Palmitic acid	HMDB0000220	C00249	Fatty acids	0.0072025	21.425
Stearic acid	HMDB0000827	C01530	Fatty acids	0.0096867	20.138
Phosphogluconic acid	HMDB0001316	C00345	Carbohydrates	4.15 × 10^−2^	43.823
Glucose-6-phosphate	HMDB0001401	C00092	Carbohydrates	0.00014815	38.293
Fructose-1-phosphate	HMDB0000399	C01094	Carbohydrates	0.0003828	3.417
Fructose-6-phosphate	HMDB0000124	C00085	Carbohydrates	0.0007741	31.112
Galacturonic acid	HMDB0002545	C00333	Carbohydrates	0.0098498	20.066
Glucose	HMDB0000122	C00031	Carbohydrates	0.014227	18.469
UMP	HMDB0000288	C00105	Pyrimidine nucleotides	4.018 × 10^−3^	63.791
Adenosine-5-monophosphate	HMDB0000045	C00020	Purine ribonucleotides	4.16× 10^−1^	43.808
Uracil	HMDB0000300	C00106	Pyrimidines	0.0021493	26.677
UDP-N-acetylglucosamine	HMDB0000290	C00043	Pyrimidine nucleotide	0.019152	17.178
Octadecanol	HMDB0002350	D01924	Fatty alcohols	1.57 × 10^−1^	48.041
Dodecanol	HMDB0011626	C02277	Fatty alcohols	0.0091442	20.389
Glutaric acid	HMDB0000661	C00489	Dicarboxylic acids	0.0002449	3.611
Creatinine	HMDB0000562	C00791	Carboxylic acids	0.0010828	29.654
Aminomalonate	HMDB0001147	C00872	Carboxylic acids	0.011284	19.476
3-aminoisobutyric acid	HMDB0003911	C05145	Carboxylic acids	0.021615	16.652
Succinic acid	HMDB0000254	C00042	Carboxylic acids	0.034452	14.628
Pantothenic acid	HMDB0000210	C00864	Secondary alcohols	0.00027704	35.575
Urea	HMDB0000294	C00086	Ureas	0.001077	29.678
Phosphoethanolamine	HMDB0000224	C00346	Phosphate esters	0.0053146	22.745
Glycolic acid	HMDB0000115	C03547	Alpha hydroxy acids	0.005771	22.387
Glutathione	HMDB0000125	C00051	Peptides	0.033934	14.694
Pyruvic acid	HMDB0000243	C00022	Keto acids	0.048561	13.137

Fold changes (FCs) described are the ratios of UVB + LA/UVB. The class, FC, and *p* value were obtained from MetaboAnalyst v6.0.

## Data Availability

Data is contained within the article.

## References

[1] Green A., Williams G., Neale R., Hart V., Leslie D., Parsons P., Marks G.C., Gaffney P., Battistutta D., Frost C. (1999). Daily sunscreen application and betacarotene supplementation in prevention of basal-cell and squamous-cell carcinomas of the skin: A randomised controlled trial. Lancet.

[2] Heck D.E., Vetrano A.M., Mariano T.M., Laskin J.D. (2003). UVB light stimulates production of reactive oxygen species: Unexpected role for catalase. J. Biol. Chem..

[3] Svobodova A., Walterova D., Vostalova J. (2006). Ultraviolet light induced alteration to the skin. Biomed. Pap. Med. Fac. Univ. Palacky. Olomouc Czech Repub..

[4] Martinez-Alvarado Y., Amezcua-Galvez E., Davila-Rodriguez J., Sandoval-Rodriguez A., Galicia-Moreno M., Almeida-Lopez M., Lucano-Landeros S., Santos A., Monroy-Ramirez H.C., Armendariz-Borunda J. (2023). Pirfenidone Protects from UVB-Induced Photodamage in Hairless Mice. Molecules.

[5] Sreekumar P.G., Ferrington D.A., Kannan R. (2021). Glutathione Metabolism and the Novel Role of Mitochondrial GSH in Retinal Degeneration. Antioxidants.

[6] Li S., Dina Kuo H.C., Wang L., Wu R., Sargsyan D., Kong A.N. (2022). UVB Drives Metabolic Rewiring and Epigenetic Reprograming and Protection by Sulforaphane in Human Skin Keratinocytes. Chem. Res. Toxicol..

[7] Hosseini M., Dousset L., Mahfouf W., Serrano-Sanchez M., Redonnet-Vernhet I., Mesli S., Kasraian Z., Obre E., Bonneu M., Claverol S. (2018). Energy Metabolism Rewiring Precedes UVB-Induced Primary Skin Tumor Formation. Cell Rep..

[8] Yang X., Wang J., Wang H., Li X., He C., Liu L. (2021). Metabolomics study of fibroblasts damaged by UVB and BaP. Sci. Rep..

[9] Zhu M., Bowden G.T. (2004). Molecular mechanism(s) for UV-B irradiation-induced glutathione depletion in cultured human keratinocytes. Photochem. Photobiol..

[10] Piao M.J., Fernando P., Kang K.A., Fernando P., Herath H., Kim Y.R., Hyun J.W. (2024). Rosmarinic Acid Inhibits Ultraviolet B-Mediated Oxidative Damage via the AKT/ERK-NRF2-GSH Pathway In Vitro and In Vivo. Biomol. Ther..

[11] Kwon K.R., Alam M.B., Park J.H., Kim T.H., Lee S.H. (2019). Attenuation of UVB-Induced Photo-Aging by Polyphenolic-Rich Spatholobus Suberectus Stem Extract Via Modulation of MAPK/AP-1/MMPs Signaling in Human Keratinocytes. Nutrients.

[12] Park H.J., Lee J.Y., Chung M.Y., Park Y.K., Bower A.M., Koo S.I., Giardina C., Bruno R.S. (2012). Green tea extract suppresses NFkappaB activation and inflammatory responses in diet-induced obese rats with nonalcoholic steatohepatitis. J. Nutr..

[13] Elias P.M., Brown B.E., Ziboh V.A. (1980). The permeability barrier in essential fatty acid deficiency: Evidence for a direct role for linoleic acid in barrier function. J. Investig. Dermatol..

[14] Hansen H.S., Jensen B. (1985). Essential function of linoleic acid esterified in acylglucosylceramide and acylceramide in maintaining the epidermal water permeability barrier. Evidence from feeding studies with oleate, linoleate, arachidonate, columbinate and alpha-linolenate. Biochim. Biophys. Acta.

[15] Ziboh V.A., Miller C.C., Cho Y. (2000). Metabolism of polyunsaturated fatty acids by skin epidermal enzymes: Generation of antiinflammatory and antiproliferative metabolites. Am. J. Clin. Nutr..

[16] Fujii M., Shimazaki Y., Muto Y., Kohno S., Ohya S., Nabe T. (2015). Dietary deficiencies of unsaturated fatty acids and starch cause atopic dermatitis-like pruritus in hairless mice. Exp. Dermatol..

[17] Bhoir S.S., Vishwapathi V., Singh K.K. (2019). Antipsoriatic potential of Annona squamosa seed oil: An in vitro and in vivo evaluation. Phytomedicine.

[18] Liu M., Li X., Chen X.Y., Xue F., Zheng J. (2015). Topical application of a linoleic acid-ceramide containing moisturizer exhibit therapeutic and preventive benefits for psoriasis vulgaris: A randomized controlled trial. Dermatol. Ther..

[19] Choi H.J., Song B.R., Kim J.E., Bae S.J., Choi Y.J., Lee S.J., Gong J.E., Lee H.S., Lee C.Y., Kim B.H. (2020). Therapeutic Effects of Cold-Pressed Perilla Oil Mainly Consisting of Linolenic acid, Oleic Acid and Linoleic Acid on UV-Induced Photoaging in NHDF Cells and SKH-1 Hairless Mice. Molecules.

[20] Lin T.K., Zhong L., Santiago J.L. (2017). Anti-Inflammatory and Skin Barrier Repair Effects of Topical Application of Some Plant Oils. Int. J. Mol. Sci..

[21] Yang B., Zhou Y., Wu M., Li X., Mai K., Ai Q. (2020). omega-6 Polyunsaturated fatty acids (linoleic acid) activate both autophagy and antioxidation in a synergistic feedback loop via TOR-dependent and TOR-independent signaling pathways. Cell Death Dis..

[22] Chang R.S., Chen C.S., Huang C.L., Chang C.T., Cui Y., Chung W.J., Shu W.Y., Chiang C.S., Chuang C.Y., Hsu I.C. (2018). Unexpected dose response of HaCaT to UVB irradiation. In Vitro Cell Dev. Biol. Anim..

[23] Mudigonda T., Dabade T.S., Feldman S.R. (2012). A review of targeted ultraviolet B phototherapy for psoriasis. J. Am. Acad. Dermatol..

[24] Masaki H., Izutsu Y., Yahagi S., Okano Y. (2009). Reactive oxygen species in HaCaT keratinocytes after UVB irradiation are triggered by intracellular Ca(2+) levels. J. Investig. Dermatol. Symp. Proc..

[25] Glady A., Tanaka M., Moniaga C.S., Yasui M., Hara-Chikuma M. (2018). Involvement of NADPH oxidase 1 in UVB-induced cell signaling and cytotoxicity in human keratinocytes. Biochem. Biophys. Rep..

[26] Hou W., Gao W., Wang D., Liu Q., Zheng S., Wang Y. (2015). The Protecting Effect of Deoxyschisandrin and Schisandrin B on HaCaT Cells against UVB-Induced Damage. PLoS ONE.

[27] Quijano C., Trujillo M., Castro L., Trostchansky A. (2016). Interplay between oxidant species and energy metabolism. Redox Biol..

[28] Koundouros N., Poulogiannis G. (2018). Phosphoinositide 3-Kinase/Akt Signaling and Redox Metabolism in Cancer. Front. Oncol..

[29] Kumar M., Bansal N. (2022). Implications of Phosphoinositide 3-Kinase-Akt (PI3K-Akt) Pathway in the Pathogenesis of Alzheimer’s Disease. Mol. Neurobiol..

[30] Gambini J., Stromsnes K. (2022). Oxidative Stress and Inflammation: From Mechanisms to Therapeutic Approaches. Biomedicines.

[31] Habib M.A., Salem S.A., Hakim S.A., Shalan Y.A. (2014). Comparative immunohistochemical assessment of cutaneous cyclooxygenase-2 enzyme expression in chronological aging and photoaging. Photodermatol. Photoimmunol. Photomed..

[32] Mammone T., Gan D., Collins D., Lockshin R.A., Marenus K., Maes D. (2000). Successful separation of apoptosis and necrosis pathways in HaCaT keratinocyte cells induced by UVB irradiation. Cell Biol. Toxicol..

[33] Gu Y., Xue F., Xiao H., Chen L., Zhang Y. (2022). Bamboo Leaf Flavonoids Suppress Oxidative Stress-Induced Senescence of HaCaT Cells and UVB-Induced Photoaging of Mice through p38 MAPK and Autophagy Signaling. Nutrients.

[34] Liu K., Zhao C., Zhang K., Yang X., Feng R., Zong Y., He Z., Zhao Y., Du R. (2024). Pilose Antler Protein Relieves UVB-Induced HaCaT Cells and Skin Damage. Molecules.

[35] Yan J., Ma L.P., Liu F., Sun B., Tian M., Lu X., Liu X.Y., Gao L., Liu Q.J. (2022). Effect of Ultraviolet B Irradiation on Melanin Content Accompanied by the Activation of p62/GATA4-Mediated Premature Senescence in HaCaT Cells. Dose Response.

[36] Rogers H.W., Weinstock M.A., Feldman S.R., Coldiron B.M. (2015). Incidence Estimate of Nonmelanoma Skin Cancer (Keratinocyte Carcinomas) in the U.S. Population, 2012. JAMA Dermatol..

[37] Kennedy L., Sandhu J.K., Harper M.E., Cuperlovic-Culf M. (2020). Role of Glutathione in Cancer: From Mechanisms to Therapies. Biomolecules.

[38] Awasthi Y.C., Ramana K.V., Chaudhary P., Srivastava S.K., Awasthi S. (2017). Regulatory roles of glutathione-S-transferases and 4-hydroxynonenal in stress-mediated signaling and toxicity. Free. Radic. Biol. Med..

[39] Lapenna D. (2023). Glutathione and glutathione-dependent enzymes: From biochemistry to gerontology and successful aging. Ageing Res. Rev..

[40] Enya S., Yamamoto C., Mizuno H., Esaki T., Lin H.K., Iga M., Morohashi K., Hirano Y., Kataoka H., Masujima T. (2017). Dual Roles of Glutathione in Ecdysone Biosynthesis and Antioxidant Function During Larval Development in Drosophila. Genetics.

[41] He F., Ru X., Wen T. (2020). NRF2, a Transcription Factor for Stress Response and Beyond. Int. J. Mol. Sci..

[42] Silvagno F., Vernone A., Pescarmona G.P. (2020). The Role of Glutathione in Protecting against the Severe Inflammatory Response Triggered by COVID-19. Antioxidants.

[43] Jones J.T., Qian X., van der Velden J.L., Chia S.B., McMillan D.H., Flemer S., Hoffman S.M., Lahue K.G., Schneider R.W., Nolin J.D. (2016). Glutathione S-transferase pi modulates NF-kappaB activation and pro-inflammatory responses in lung epithelial cells. Redox Biol..

[44] Xiong Y., Uys J.D., Tew K.D., Townsend D.M. (2011). S-glutathionylation: From molecular mechanisms to health outcomes. Antioxid. Redox Signal.

[45] Buckman S.Y., Gresham A., Hale P., Hruza G., Anast J., Masferrer J., Pentland A.P. (1998). COX-2 expression is induced by UVB exposure in human skin: Implications for the development of skin cancer. Carcinogenesis.

[46] Manosalva C., Alarcon P., Gonzalez K., Soto J., Igor K., Pena F., Medina G., Burgos R.A., Hidalgo M.A. (2020). Free Fatty Acid Receptor 1 Signaling Contributes to Migration, MMP-9 Activity, and Expression of IL-8 Induced by Linoleic Acid in HaCaT Cells. Front. Pharmacol..

[47] Kim M., Gu G.J., Koh Y.S., Lee S.H., Na Y.R., Seok S.H., Lim K.M. (2018). Fasiglifam (TAK-875), a G Protein-Coupled Receptor 40 (GPR40) Agonist, May Induce Hepatotoxicity through Reactive Oxygen Species Generation in a GPR40-Dependent Manner. Biomol. Ther..

[48] Mercola J., D’Adamo C.R. (2023). Linoleic Acid: A Narrative Review of the Effects of Increased Intake in the Standard American Diet and Associations with Chronic Disease. Nutrients.

[49] Sun C., Li Y., Li X., Sun J. (2020). Agonism of Gpr40 Protects the Capacities of Epidermal Stem Cells (ESCs) Against Ultraviolet-B (UV-B). Drug Des. Devel Ther..

[50] Boukamp P., Petrussevska R.T., Breitkreutz D., Hornung J., Markham A., Fusenig N.E. (1988). Normal keratinization in a spontaneously immortalized aneuploid human keratinocyte cell line. J. Cell Biol..

[51] Geng R., Kang S.G., Huang K., Tong T. (2023). alpha-Ionone protects against UVB-induced photoaging in epidermal keratinocytes. Chin. Herb. Med..

[52] Masuma R., Kashima S., Kurasaki M., Okuno T. (2013). Effects of UV wavelength on cell damages caused by UV irradiation in PC12 cells. J. Photochem. Photobiol. B.

[53] Alarcon-Gil J., Sierra-Magro A., Morales-Garcia J.A., Sanz-SanCristobal M., Alonso-Gil S., Cortes-Canteli M., Niso-Santano M., Martinez-Chacon G., Fuentes J.M., Santos A. (2022). Neuroprotective and Anti-Inflammatory Effects of Linoleic Acid in Models of Parkinson’s Disease: The Implication of Lipid Droplets and Lipophagy. Cells.

[54] Kadotani A., Tsuchiya Y., Hatakeyama H., Katagiri H., Kanzaki M. (2009). Different impacts of saturated and unsaturated free fatty acids on COX-2 expression in C(2)C(12) myotubes. Am. J. Physiol. Endocrinol. Metab..

[55] Masner M., Lujea N., Bisbal M., Acosta C., Kunda P. (2021). Linoleic and oleic acids enhance cell migration by altering the dynamics of microtubules and the remodeling of the actin cytoskeleton at the leading edge. Sci. Rep..

[56] Nava Lauson C.B., Tiberti S., Corsetto P.A., Conte F., Tyagi P., Machwirth M., Ebert S., Loffreda A., Scheller L., Sheta D. (2023). Linoleic acid potentiates CD8(+) T cell metabolic fitness and antitumor immunity. Cell Metab..

[57] Serna-Marquez N., Villegas-Comonfort S., Galindo-Hernandez O., Navarro-Tito N., Millan A., Salazar E.P. (2013). Role of LOXs and COX-2 on FAK activation and cell migration induced by linoleic acid in MDA-MB-231 breast cancer cells. Cell. Oncol..

[58] Fiehn O. (2016). Metabolomics by Gas Chromatography-Mass Spectrometry: Combined Targeted and Untargeted Profiling. Curr. Protoc. Mol. Biol..

[59] Livak K.J., Schmittgen T.D. (2001). Analysis of relative gene expression data using real-time quantitative PCR and the 2(-Delta Delta C(T)) Method. Methods.

